# Enhancing quality and climate resilient traits in vegetatively propagated polyploids: transgenic and genome editing advancements, challenges and future directions

**DOI:** 10.3389/fgene.2025.1599242

**Published:** 2025-08-11

**Authors:** Surya Krishna Sakthivel, Amaranatha Reddy Vennapusa, Kalpalatha Melmaiee

**Affiliations:** Department of Agriculture and Natural Resources, Delaware State University, Dover, DE, United States

**Keywords:** polyploids, vegetative propagation, genome editing, transgenics, climate resilience, quality, food security

## Abstract

Vegetatively propagated polyploid crops such as potato, strawberry, sugarcane, and banana play a crucial role in global agriculture by meeting essential nutritional and food demands. The quality of the economically important traits in these crops is significantly affected by global climate change. However, their complex genomes and clonal propagation nature pose significant challenges for traditional breeding to improve quality and climate-resilient traits. Transgenics and genome editing offer promising solutions in crop improvement to enhance yield, quality, and biotic and abiotic stress tolerance. Despite these advancements, several challenges persist, such as a lack of genotype-independent transformation protocols, random transgene integration, unintended mutations, and somaclonal variation. The complexity of polyploid genomes also necessitates optimizing editing tools to improve precision and efficiency. Regulatory hurdles and public acceptance further influence the commercial success of genetically engineered crops. Employing efficient transgene-free genome-editing platforms can help to overcome the regulatory hurdles and accelerate breeding even in heterozygous backgrounds. This review reports the recent progress, obstacles, and prospects of transgenics and genome editing in vegetatively propagated crops, namely, potato, strawberry, banana, and sugarcane, focusing on quality and climate-resilient traits and methods to address technical challenges and navigate regulatory hurdles. The reported advancements in genetic engineering approaches for addressing challenges in improving the vegetatively propagated polyploid crops have tremendous potential in ensuring food security and agricultural sustainability in the face of climate change.

## 1 Introduction

Polyploidy is one of the most important forces of evolution and speciation in plants (Heslop-Harrison et al., 2023). Polyploids have more than two sets of chromosomes in their genome, and based on their origin, polyploids are divided into two distinct categories: autopolyploid and allopolyploid. In general, autopolyploids, such as potato (*Solanum tuberosum* L.) and bananas (*Musa* spp.), contain multiple sets of the same chromosomes originating from the same species as a result of intraspecific genome duplication (Sattler et al., 2016; Heslop-Harrison et al., 2023). In contrast, allopolyploids have multiple sets of the same chromosomes derived from different species of the same genus through hybridization and subsequent genome duplication (Sattler et al., 2016; Heslop-Harrison et al., 2023). Among cultivated polyploids, many of them are allopolyploids, including wheat (*Triticum aestivum*), sugarcane (*Saccharum spp*), strawberry (*Fragaria × ananassa*), soybean (*Glycine max*), grape (*Vitis vinifera)*, apple (*Malus domestica*), and many others.

Polyploids exhibit complex genetic architecture and inheritance compared to diploids. They are characterized by high gene copy numbers, heterozygosity, genome size, gene redundancy, homoeologous recombination, repetitive sequences, complex meiotic behaviors, dosage effects, and altered gene expression patterns (Birchler et al., 2005; Comai, 2005; Osabe et al., 2012; Te Beest et al., 2012; Meirmans et al., 2018; Soares et al., 2021; Healey et al., 2024). The complex genetics of polyploids pose challenges in the varietal development process for breeding new cultivars, and common problems include infertility, hybrid sterility, and inbreeding depression (Levin, 2002; Comai, 2005; Paterson, 2005; Udall and Wendel, 2006; Sattler et al., 2016; Bradshaw, 2024).

Despite breeding difficulties, polyploids have numerous advantages in adaptation and biomass production compared to diploids (Udall and Wendel, 2006; Osabe et al., 2012; Sattler et al., 2016). High heterozygosity and genetic variability make polyploids more vigorous and improve their buffering capacity in response to various biotic and abiotic stresses compared to their diploid counterparts (Adams and Wendel, 2005; Van de Peer et al., 2009). Most polyploids have both asexual and sexual modes of reproduction, which enable the indefinite multiplication of transgressive segregants with desired characteristics obtained through hybridization between different parental lines (Chahal and Gosal, 2002; Comai, 2005; Osabe et al., 2012; Sattler et al., 2016; Schiessl et al., 2019). Vegetative propagation is the primary mode of reproduction widely used to cultivate polyploid crops such as potato, sugarcane, strawberry, banana, and apple. This allows these crops to maintain favorable heterozygosity and pass on hybrid superiority for many generations, making it easier to maintain true-to-type plants (Comai, 2005; Udall and Wendel, 2006). It is interesting to note that most vegetatively propagated polyploids show high levels of outcrossing and exhibit a perennial nature (McKey et al., 2010).

Classical breeding and marker-assisted breeding have played significant roles in improving yield, quality, and stress resistance in vegetatively propagated polyploid crop plants (Collard and Mackill, 2008; Bharadwaj, 2015; Sattler et al., 2016; Wolter et al., 2019). Although classical breeding is highly appealing, it is time-consuming and resource-intensive (Acquaah, 2015; Wolter et al., 2019). Additionally, factors such as self-incompatibility, hybrid sterility, infertility, and limited availability of variation and novel alleles for traits of interest in the natural gene pool severely constrain clonal crop improvement through traditional and marker-assisted breeding (Acquaah, 2015; Bharadwaj, 2015). Transgenic-based breeding and genome editing speed up crop improvement by introducing novel genes from different organisms and creating new alleles for existing traits, respectively, which are highly challenging to achieve using conventional techniques (Kamthan et al., 2016; Gao, 2021; Marone et al., 2023).

**FIGURE 1:**
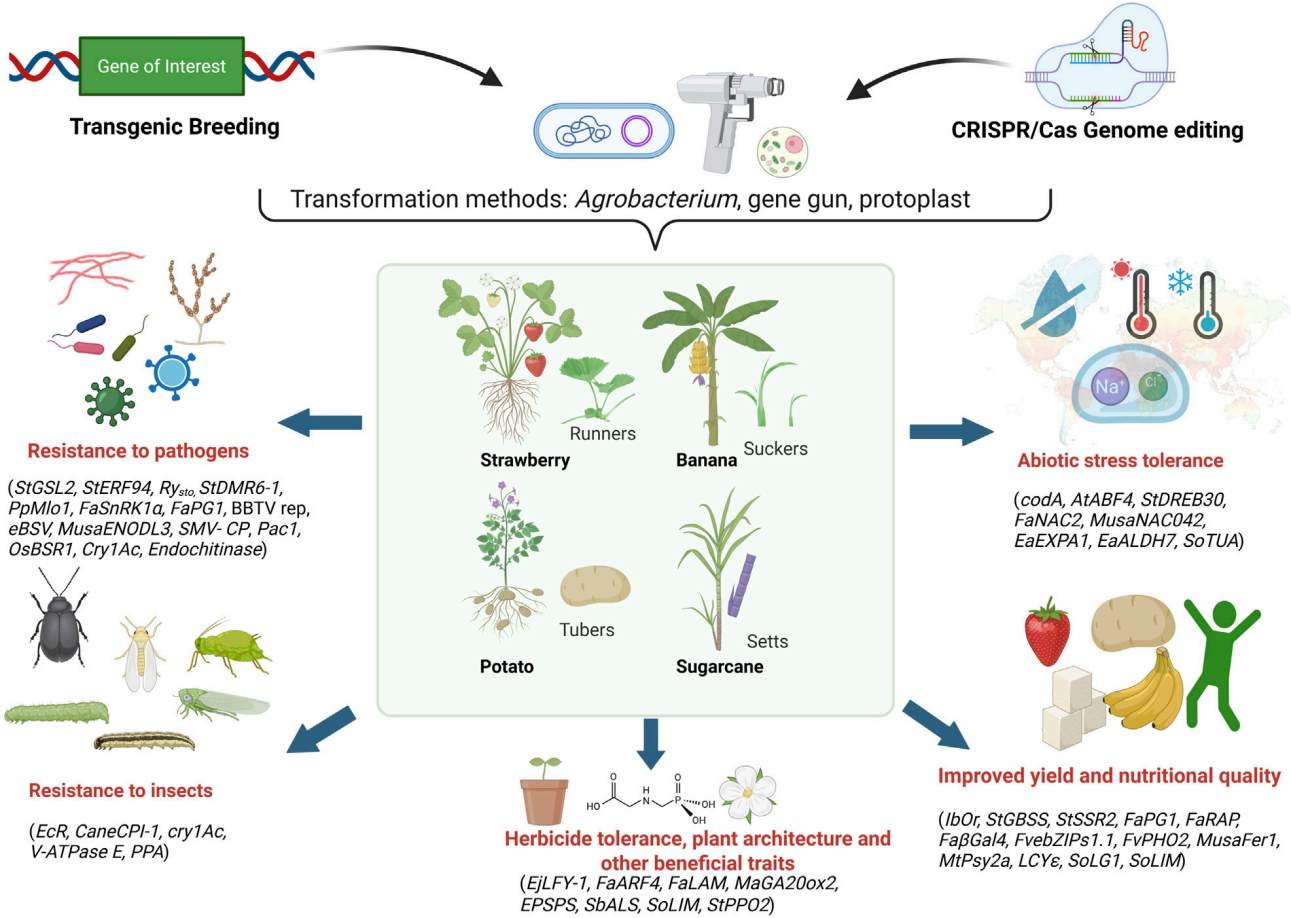
Application of transgenics and genome editing for improving abiotic stress tolerance, biotic stress resistance, yield, quality and other beneficial traits in vegetatively propagated polyploids by targeting genes of interest. Runners, suckers, tubers, and setts represent the commercial propagation materials of strawberry, banana, potato, and sugarcane, respectively Image created using Biorender (https://BioRender.com).

Climate change profoundly impacts crop production worldwide, with developing countries particularly vulnerable to its adverse effects (Kogo et al., 2021; Malhi et al., 2021; Yuan et al., 2024). The shifting precipitation patterns, rising temperatures, and increased frequency of extreme weather events such as unprecedented rainfall and severe droughts are significantly affecting crop production and threatening global food security (Kogo et al., 2021; Malhi et al., 2021; Yuan et al., 2024). These changes also facilitate the emergence and spread of new virulent strains of plant pathogens and insect pests, further exacerbating the situation (Malhi et al., 2021; Skendžić et al., 2021; Singh et al., 2023). Whereas several research and review articles have individually described transgenic and gene editing events in the context of climate resilience and quality related traits in vegetatively propagated crops (Tripathi L. et al., 2019; Schaart et al., 2021; Tiwari et al., 2022; Lakhani et al., 2023; Surya Krishna et al., 2023a; Vondracek et al., 2024). However, there is a lack of comprehensive review addressing both approaches collectively. The current review aims to bridge this existing gap by providing an integrated overview of both transgenic and gene editing studies relevant to these crops. The current review cited the literature from 2002 to 2025 based on articles, book chapters, and websites. The information was sourced from Google Scholar, PubMed, Web of Science, and other databases. The listed transgenic events and gene-edited lines provided based on literature between 2011 and 2025. This review highlights the importance of transgenics and genome editing approaches, mainly CRISPR/Cas (Clustered Regularly Interspaced Short Palindromic Repeats/CRISPR-associated proteins), in developing climate-resilient and quality-enhanced cultivars in the era of climate change, focusing on four major clonally propagated polyploid crops: potato, banana, strawberry, and sugarcane (Figure 1).

## 2 Potato

Potato (*Solanum tuberosum* L.) is the third most important food crop in the world, following wheat and rice, and is consumed widely across the globe (Halterman et al., 2016; del Mar Martínez-Prada et al., 2021). Significant progress has been made in developing transgenic and genome-edited potatoes with improved yield, pest and disease resistance, and enhanced quality (Nahirñak et al., 2022). In potato, the transformation efficiency was reported from 3% to 60%, which is highly dependent on type of genotype, explant and transformation method (Craze et al., 2018; Nahirñak et al., 2022). *Agrobacterium*-mediated transformation is the most commonly used method in potato genetic engineering (Craze et al., 2018; Nahirñak et al., 2022). Since 1995, various transgenic potato events have been developed for commercial purposes. First, Monsanto introduced the transgenic potato, NewLeaf, which conferred resistance to the Colorado potato beetle through the introduction of the *cry3A* gene in the U.S. and Canada (Halterman et al., 2016; del Mar Martínez-Prada et al., 2021). By 1998, enhanced versions of NewLeaf were created by stacking additional transgenes, such as *PLRV replicase*, *Helicase*, and *PVY coat protein*, providing resistance to potato leafroll virus and potato virus Y, alongside the *cry3A* gene (Halterman et al., 2016; del Mar Martínez-Prada et al., 2021).

Amylopectin from potatoes has various applications in bioplastic, textile, and ethanol industries; separating it from the amylose adds more processing cost, and chemical treatments lead to environmental pollution. Techniques such as RNA interference (RNAi) and CRISPR/Cas9 have successfully silenced or knocked out the *granule-bound starch synthase* (GBSS) genes responsible for amylose production, enabling the development of amylose-free potatoes for industrial applications (Brummell et al., 2015; Andersson et al., 2017; 2018; Kusano et al., 2018). Acrylamide, a carcinogenic compound formed from a reaction between natural sugars and the amino acid asparagine during high-temperature cooking (e.g., frying, roasting, or baking), poses health risks (Bethke and Bussan, 2013). Using RNAi, Zhu et al. (2014) silenced the *vacuolar invertase (VInv)* gene to develop low-acrylamide potatoes by lowering the reducing sugar formation in the tubers and making potatoes more suitable for French fries. In 2015, the U.S. approved the commercialization of J.R. Simplot’s Innate^®^ 1.0 potatoes, which exhibit reduced acrylamide formation and black spot bruising through the downregulation of *Asn1* and *Ppo2* genes (del Mar Martínez-Prada et al., 2021; ISAAA, 2025). By 2017, Innate^®^ 2.0 potatoes further incorporated traits such as resistance to late blight pathogens, reduced bruising and asparagine content, and enhanced cold storage ability (del Mar Martínez-Prada et al., 2021; ISAAA, 2025).

CRISPR/Cas-mediated genome editing has also significantly advanced potato improvement. For instance, the knockout of the *St16DOX* gene eliminates toxic steroidal glycoalkaloids (Nakayasu et al., 2018). CRISPR/Cas9-mediated knockout of an S gene, *StPM1* gene in potato enhanced the resistance to Phytophthora infection without affecting growth and development (Bi et al., 2023). *StPM1* negatively regulates plant immunity against invading pathogens by aiding vacuolar-mediated degradation of StRbohC, an NADPH oxidase that is involved in oxidative burst under infection conditions (Bi et al., 2023). Furthermore, the knockout of *StNPR3* gene showed resistance to potato zebra chip disease through salicylic acid (SA) mediated defense and jasmonic acid (JA) catabolism (Ramasamy et al., 2024). *StNPR3*-edited lines showed higher expression of marker genes for plant defense such as *NPR1*, *WRKY6*, *PR1* and *PR3* and exhibited enhanced SA accumulation under both uninfected and infected conditions (Ramasamy et al., 2024). Veillet et al. (2019) employed cytidine base editors to modify *ALS* genes, further demonstrating the efficacy of base editing in potatoes. A recent review by Kumari et al. (2024), specifically focused on genome editing in potato, provides detailed information on the current status and applications of genome editing in this crop. We provided an overall list of events related to quality and stress tolerance in Table 1 which highlights the application of transgenics and genome editing techniques for potato crop improvement.

**TABLE 1:** Application of biotechnological approaches for potato crop improvement.

S. No	Genetic engineering approach	Gene of interest	Improved trait	References
1	Transgenics	Coat protein of potato virus Y, potato leafroll virus, potato virus A	Resistance against potato virus Y (PVY), potato leafroll virus, and potato virus A	Chung et al. (2013)
2	Transgenics	*CodA*	Enhanced drought tolerance and recovery ability	Cheng et al. (2013)
3	Transgenics	*StGSL2*	Resistance to blackleg disease	Mohan et al. (2014)
4	Transgenics	*AtLecRK-I.9*	Enhanced resistance *to P. infestans*	Bouwmeester et al. (2014)
5	Transgenics	*AtABF4*	Increased tuber yield, improved storage capability and processing quality of the tubers, and enhanced salt and drought tolerance	Muñiz García et al. (2018)
6	Transgenics	*STANN1*	Tolerance to high light and drought stresses	Szalonek et al. (2015)
7	Transgenics	*IbOr*	Increased accumulation of carotenoid, oxidative stress tolerance	Goo et al. (2015)
8	Transgenics	*StGLP*	Enhanced thermo-tolerance	Gangadhar et al. (2021)
9	Transgenics	*StnsLTP1*	Enhanced tolerance to heat, drought, and salt	Gangadhar et al. (2016)
10	Transgenics	*PaSOD, RaAPX*	Increased salt tolerance and starch accumulation	Shafi et al. (2017)
11	Transgenics	*AtPDX-II*	Enhanced accumulation of vitamin B6 in tuber tissues and tolerance to abiotic stresses	Bagri et al. (2018)
12	Transgenics	*StERF94*	Improved Resistance to *Fusarium solani*	Charfeddine et al. (2019)
13	Transgenics	*RB, Rpi-blb2* and *Rpi-vnt1.1*	Enhanced resistance to *P. infestans*	Ghislain et al. (2019)
14	Transgenics	*Ry*_*sto*_	Resistance against potato virus Y	Grech-Baran et al. (2020)
15	Transgenics	*StSOD1*	Increased low temperature tolerance	Che et al. (2020)
16	Transgenics	*CP4-EPSPS*	Resistance against glyphosate herbicide	Bakhsh et al. (2020)
17	Transgenics	*StDREB30*	Enhanced salt and drought stress tolerance	Ul Ain-Ali et al. (2024)
18	Transgenics	*StDREB1*	Improved salt and drought stress tolerance	Bouaziz et al. (2013)
19	Transgenics	*Rice cystatin OCI and OCII*	Enhanced resistance against colorado potato beetle	Cingel et al. (2014)
20	Transgenics	*Molting-associated EcR gene*	Enhanced resistance against Colorado potato beetle	Hussain et al. (2019)
21	CRIPSR/Cas9	*StGBSS*	Low amylose starch in their tubers	Kusano et al. (2018)
22	CRIPSR/Cas9	*St16DOX*	Steroidal glycoalkaloids free hairy roots	Nakayasu et al. (2018)
23	CRIPSR/Cas9	*StGBSS*	Amylose free starch in their tubers	Johansen et al. (2019)
25	CRIPSR/Cas9	*S-RNase*	Self-compatible mutants	Enciso-Rodriguez et al. (2019)
26	CRIPSR/Cas9	*StPPO2*	Reduced enzymatic browning in tubers	González et al. (2020)
27	CRIPSR/Cas9	*StDND1, StCHL1, and StDMR6-1*	Increased resistance against late blight	Kieu et al. (2021)
28	CRIPSR/Cas9	*StSSR2*	Reduction in steroidal glycoalkaloids content	Zheng et al. (2021)
29	CRIPSR/Cas9	*StERF3*	Enhanced resistance to the late blight disease	Razzaq et al. (2022)
30	CRIPSR/Cas9	*eIF4E1*	Reduced PVY infection	Lucioli et al. (2022)
31	CRIPSR/Cas9	*StNRL1*	Increased resistance to late blight and susceptibility to early blight in potato and enhanced tolerance to drought and Salinity stress	Norouzi et al. (2024)
32	CRIPSR/Cas9	*StPM1*	Enhanced potato tolerance to late blight	Bi et al. (2023)
33	CRIPSR/Cas9	*StDMR6-1*	Increased resistance to late blight, early blight, and common scab	Karlsson et al. (2024)

## 3 Strawberry

Strawberry is one of the economically important fruit crops, widely grown and consumed in different parts of the world for its organoleptic properties and nutritional value (Biswas et al., 2019). Cultivated strawberry (*Fragaria × ananassa*) is an allo-octoploid (2n = 8x = 56) originated from the interspecific hybridization between diploid progenitors: *Fragaria vesca* and *Fragaria iinumae* (Jin et al., 2023; Song et al., 2024). Due to the high ploidy level and heterozygous nature, breeding strawberries through classical methods for targeted trait improvement is a daunting task. Overexpressing or functional downregulation of a gene of interest for a particular trait improvement has been more promising compared to classical breeding methods, and the desired transgenic events or mutants can be multiplied using either runners or through tissue culture (López-Casado et al., 2023; Zhang et al., 2023; Luo et al., 2024). In addition to crop improvement, genetic engineering approaches also serve as an efficient tool for validating the QTLs or genes identified through genomics and transcriptomics.

Transgenics have been successfully employed for quality improvement and disease resistance in strawberry (Wang Y. et al., 2017; Li et al., 2021; Zhang et al., 2021; Luo et al., 2024). Agrobacterium-mediated transformation is commonly used for both the development of transgenic events and genome-edited (GE) lines, with an efficiency ranging between 2.9%–100% depending on the genotype and explants used for genetic transformation (Schaart, 2014; Vondracek et al., 2024). Transgenic plants overexpressing *miR399a* showed increased phosphorus uptake and improved total sugar, soluble solid, and vitamin C contents (Wang Y. et al., 2017). The fungal diseases, gray mold caused by *Botrytis cinerea* and anthracnose by *Colletotrichum* species, are the devastating fruit rot diseases in strawberry that severely affect fruit yield and quality (Petrasch et al., 2019; Ji et al., 2022). Zhang et al. (2021) reported that overexpression of *FaMAPK5* and *FaMAPK10* genes showed increased resistance to gray mold fungus and enhanced production of antioxidants. *FaSnRK1α* gene overexpression in strawberry plants induced the expression of SA biosynthetic genes, *FaPAL1* and *FaPAL2*, which helped to obtain the elevated levels of SA and thus led to the enhanced resistance to *B. cinerea* (Luo et al., 2024). Further, Under *B. cinerea* infection, *FaSnRK1α*-OE lines had increased concentrations of SA allowing *FaSnRK1α* to interact with *TGA* and *WRKY33.2* transcription factors to induce the expression of pathogen-related genes, namely, *PR1.1, FaPR1.2, FaPR1.5, FaPR4.2, FaPR4.3*, and *FaPR5*, which ultimately improved fruit resistance to *B. cinerea* (Luo et al., 2024). In addition to biotic stresses, strawberry production is significantly affected by abiotic stresses including, extreme temperatures, drought, and salinity, that affect growth, physiology, fruit yield, and quality (Ghaderi et al., 2018; Menzel, 2021; Ullah et al., 2024; Rodríguez-Aguirre et al., 2025). Heterologous expression of *At-rty* improved drought stress tolerance in strawberry due to accumulation of indolylacetic acid (IAA) and abscisic acid (ABA) (Li et al., 2021). The transgenic lines showed improved water use efficiency and reduced water loss along with the enhanced activity of antioxidants under drought conditions (Li et al., 2021).

Despite the octoploid nature of the cultivated strawberry, genome editing has also shown some significant success in enhancing disease resistance and altering the fruit properties. For example, CRISPR/Cas9 induced *PG1* mutants in octoploid strawberry have shown less *B. cinerea* infection, increased fruit firmness, and altered fruit shape (López-Casado et al., 2023). Martín-Pizarro et al. (2019) edited the *FaTM6* genes using CRISPR/Cas9 GE to study the role of *TM6 MADS-box* gene in the octoploid strawberry. The mutants showed abnormal flower morphology, decreased pollen production, and receptacle growth. CRISPR/Cas9 GE has been effectively used to manipulate the coloration of strawberry fruits. The RAP gene plays a crucial role in anthocyanin formation in strawberries, which gives the characteristic red color (Luo et al., 2018; Gao et al., 2020). It encodes a glutathione S-transferase (GST) protein that facilitates the transport of anthocyanins from the cytosol to the vacuole, where they accumulate to give the fruit its red color (Luo et al., 2018; Gao et al., 2020). The knockout of multiple copies of *FaRAP* gene has produced white colored strawberry fruits (Gao et al., 2020). The application of transgenics and genome editing techniques for strawberry crop improvement is given in Table 2. The recent reviews focused on strawberry biotechnology programs, genomic resources, transgenics, and genome editing have been listed and discussed, along with the prerequisites for crop improvement (Mukherjee and Gantait, 2024; Vondracek et al., 2024). Genetic engineering and genome editing have huge potential in improving strawberry crop production by enhancing fruit quality, producing novel fruit types, and developing stress-resilient strawberry cultivars.

**TABLE 2:** Transgenics and genome editing for strawberry crop improvement.

S. No.	Genetic engineering technique	Gene of interest	Improved trait	References
1	Transgenics	*FaPG1*	Enhanced tissue integrity, and firmness in ripe strawberry fruits	Posé et al. (2013)
2	Transgenics	*PpMlo1*	Resistance to *Fragaria*-specific powdery mildew	Jiwan et al. (2013)
3	Transgenics	*Trichoderma harzianum bgn13.1*	Increased tolerance to crown rot	Mercado Carmona et al. (2015)
4	Transgenics	*AtNPR1*	Broad-spectrum disease resistance	Silva et al. (2015)
5	Transgenics	*AtELP3/ELO3, ELP4/ELO1*	Enhanced disease resistance to anthracnose crown rot, powdery mildew, and angular leaf spot	Silva et al. (2017)
6	Transgenics	*EjLFY-1*	Promoted early flowering	Liu Y. et al. (2017)
7	Transgenics	*RdreB1BI*	Enhanced drought tolerance	Gu et al. (2017)
8	Transgenics	*miR399*	Improved fruit quality	Wang Y. et al. (2017)
9	Transgenics	*FaβGal4*	Increased cell wall galactose levels and reduced fruit softening	Paniagua et al. (2016)
10	Transgenics	*FaNAC2*	Better performance under salt, cold, and drought stress	Liang et al. (2020)
11	Transgenics	*FaARF4*	Early flowering	Dong et al. (2021)
12	Transgenics	*MANNOSE-BINDING LECTIN 1 (MBL1)*	Tolerance to *Colletotrichum fioriniae* and *Botrytis cinerea*	Ma et al. (2023)
13	Transgenics	*FaRGLyase1*	Increased firmness of ripe fruits	Ric-Varas et al. (2024)
14	Transgenics	*FaERF2*	Resistance to *B. cinerea*	Peng et al. (2024)
15	Transgenics	*FaSnRK1α*	Resistance to *B. cinerea*	Luo et al. (2024)
16	CRISPR/Cas9	*FaRAP*	White fruits	Gao et al. (2020)
17	CRISPR/Cas9	*FvebZIPs1.1*	Increased sugar content	Xing et al. (2020)
18	CRISPR/Cas9	*FveRGA1 and FveARF8*	Increased fruit size	Zhou et al. (2021)
19	CRISPR/Cas9	*FaLAM*	Reduction in runner production	Feng et al. (2021)
20	CRISPR/Cas9	*FaPG1*	Increased fruit firmness and reduced *B. cinerea* infection	López-Casado et al. (2023)
21	CRISPR/Cas9	*FvPHO2*	Increased phosphorus, anthocyanin, and soluble solid content in fruits	Zhang et al. (2023)
22	CRISPR/Cas9	*FvWRKY50*	Accelerated flowering time, delayed leaf senescence, and anthocyanin accumulation in fruits	Chen et al. (2023)

## 4 Banana

Banana (*Musa* spp.) is one of the important fruit crops consumed globally from the tropics to temperate regions and plays a significant role in satisfying the nutrient needs of people (Tripathi et al., 2024b). Most edible are diploid or triploid hybrids from *Musa acuminata* (A-genome) alone or from hybridization with *Musa balbisiana* (B-genome) (Perrier et al., 2011). Most commercial cultivars are seedless triploids, such as the commercially important Cavendish dessert banana (AAA) and the staple cooking African plantains (AAB) (Sardos et al., 2016). The gametic sterility, coupled with selection for edible pulp enhancement, led to parthenocarpic fruits during the domestication process (Perrier et al., 2011; Sardos et al., 2016). Bananas are propagated primarily through suckers and the meristem tip culture. Meristem tip culture produces clean seed material without any disease infection (Saraswathi et al., 2024).

Despite their major role in food security, bananas are one of the least genetically improved crops through breeding, due to their parthenocarpic nature, sterility, heterozygosity, and polyploid nature (Nansamba et al., 2020; Ganapathi et al., 2021; Tripathi et al., 2024b). In this scenario, genetic engineering offers endless opportunities for their crop improvement (Ganapathi et al., 2021; Tripathi et al., 2024b). Transgenic bananas have been effective against various biotic and abiotic stresses. Agrobacterium-mediated transformation is a majorly reported method for banana genetic modification due to its advantages of stable integration and lower copy number insertions (Cheng et al., 2024). Transformation efficiencies was ranging from as low as 2% to as high as 100%, depending on the explant, transformation method, and genotype (Liu J. et al., 2017; Dong et al., 2020). RNAi transgenic bananas expressing *acetylcholinesterase* genes from the aphid *Pentalonia nigronervosa*, showed reduced banana aphid infestation (Jekayinoluwa et al., 2021). Banana aphids are the vectors of banana bunchy top virus (BBTV), the causal agent of banana bunchy top disease. Besides BBTV, one of the devastating diseases of banana is fusarium wilt caused by the fungus, *Fusarium oxysporum* f. sp. *Cubense*. Transgenic lines overexpressing *Ced9* anti-apoptosis gene derived from the nematode *Caenorhabditis elegans* (Paul et al., 2011) and *RGA2* (Dale et al., 2017), a putative nucleotide-binding and leucine-rich repeat (NB-LRR)-type resistance (R) gene, from a seedling of *Musa acuminata* ssp. *malaccensis* showed resistance to fusarium wilt disease. On the other hand, banana transgenic lines have been very successful in combating abiotic stresses. Overexpression of aquaporin genes such as *MusaPIP1;2* and *MaPIP2-7* exhibited an improved stress tolerance to various abiotic stresses such as drought, cold, and salinity (Xu et al., 2020; 2021). The transgenic lines achieved enhanced stress tolerance and exhibited elevated proline, soluble sugar, chlorophyll, K^+^/Na^+^ ratio, and ABA content with lower ion leakage and malondialdehyde (MDA) content under stress conditions (Xu et al., 2020; 2021). Genetic engineering has not only proven to be effective against biotic and abiotic stresses but also improved nutritional content. For example, golden bananas developed through the expression of a *Fe’i banana-derived phytoene synthase 2a* (*MtPsy2a*) gene and *maize phytoene synthase 1* (*ZmPsy1*) gene showed elevated pro-vitamin A in the fruits under field conditions (Paul et al., 2017).

Genome editing has been applied for creating stress-tolerant mutants and functional genomics studies in banana crop improvement programs. Mutating downy mildew resistance 6 (DMR6), a susceptibility gene encoding 2-oxoglutarate Fe(II)-dependent oxygenase (2OGO), induced the resistance to Banana Xanthomonas Wilt (BXW), bacterial disease, under field conditions without showing any off-target effects (Tripathi et al., 2021). *DMR6* acts as a suppressor of plant immunity and is upregulated under pathogen infection (Tripathi et al., 2021). CRISPR/Cas9-based genome-editing technology was applied to inactivate endogenous *banana streak virus* (eBSV) in the B genome of plantain (AAB), and these GE lines showed 75% less viral symptoms under water stress conditions in comparison to the control plants, indicating the inactivation of eBSV in GE lines (Tripathi J. N. et al., 2019). CRISPR/Cas9 GE was used to develop the β-carotene-enriched banana cultivar by mutating the fifth exon of the *lycopene epsilon-cyclase (LCYε)* gene (Kaur et al., 2020). Metabolic profiling of the GE fruits showed enhanced accumulation of β-carotene content up to 6-fold, suggesting the potential of CRISPR technology in improving the nutritional quality of the bananas without the aid of transgenes (Kaur et al., 2020). Some of the recent reviews primarily focused on banana improvement through biotechnological applications, providing an elaborate discussion on banana genetic resources and genetic engineering (Cheng et al., 2024; Tripathi et al., 2024b). Table 3 highlights the application of transgenics and genome editing techniques for banana crop improvement.

**TABLE 3:** Banana crop improvement through advanced biotechnological approaches.

S. No	Genetic engineering technique	Gene of interest	Improved trait	References
1	Transgenics	BBTV rep	Resistance against Banana bunchy top virus	Elayabalan et al. (2013)
2	Transgenics	*PhDef1* and *PhDef2*	Resistance against *Fusarium oxysporum race 1*	Ghag et al. (2014)
3	Transgenics	*OsXa21*	Resistance against *Xanthomonas campestris pv. musacearum*	Tripathi et al. (2014)
4	Transgenics	*Maize Cystine inhibitor*	Resistance against *Radopholus similis* and *Helicotylenchus multicinctus*	Tripathi et al. (2015)
5	Transgenics	*Hrap* and *Pflp*	Resistance against *X. campestris pv. musacearum*	Tripathi et al. (2017)
6	Transgenics	*RGA2* and *Ced9*	Resistance against *Fusarium oxysporum cubense* tropical race 4	Dale et al. (2017)
7	Transgenics	*AtBAG4*	Resistance against *Fusarium oxysporum cubense* race 1	Umesha et al. (2025)
8	Transgenics	*MusaFer1*	Enhanced oxidative stress tolerance and improved iron content	Yadav et al. (2017)
9	Transgenics	*MtPsy2a*	Elevated pro-vitamin A	Paul et al. (2017)
10	Transgenics	*ZmPsy1*	Elevated pro-vitamin A	Paul et al. (2017)
11	Transgenics	*MusaNAC042*	Improved abiotic stress tolerance	Tak et al. (2017)
12	Transgenics	*AhcAPX*	Improved abiotic stress tolerance	Shekhar et al. (2019)
13	Transgenics	*MusaPIP1;2*	Improved abiotic stress tolerance	Sreedharan et al. (2013)
14	Transgenics	*MaPIP1;1*	Improved abiotic stress tolerance	Xu et al. (2021)
15	CRISPR/Cas9	*eBSV*	Banana streak virus resistance	Tripathi J. N.et al. (2019)
16	CRISPR/Cas9	*LCYε*	Increased Beta Carotene content in fruits	Kaur et al. (2020)
17	CRISPR/Cas9	*MaGA20ox2*	Semi-dwarf plant type	Shao et al. (2019)
18	CRISPR/Cas9	*DMR6*	Resistance against *X. campestris pv. musacearum*	Tripathi et al. (2021)
19	CRISPR/Cas9	*MusaENODL3*	Resistance against *X. campestris pv. musacearum*	Ntui et al. (2024)

## 5 Sugarcane

Sugarcane is an important crop primarily cultivated in the tropical and subtropical regions of the world (Dinesh Babu et al., 2022; Surya Krishna et al., 2023a). It plays a crucial role in satisfying global sugar demands, accounting for 80% of global sugar production (Dinesh Babu et al., 2022). Beyond sugar production, sugarcane is the major raw material for bioethanol production, a cleaner alternative to fossil fuels, and byproducts such as molasses and bagasse, which are used in various industries ranging from alcohol production to electricity generation (Surya Krishna et al., 2023a). Sugarcane possesses a highly complicated genetic architecture that makes crop improvement and genetic studies challenging. Unlike other cultivated polyploids, sugarcane is an aneuploid with an interspecific origin, with chromosomes ranging from 80 to 130 (Vieira et al., 2018; Piperidis and D’Hont, 2020; Healey et al., 2024). Modern cultivars (*Saccharum* spp.) are interspecific hybrids with tolerance to the aneuploid constitution, producing offspring with unique chromosome combinations when propagated through seeds (Vieira et al., 2018; Healey et al., 2024). In this context, genetic engineering techniques offer a unique advantage by enabling the introduction or modification of genes for specific traits in superior clones without altering other traits, a process that is highly challenging to achieve through conventional or marker-assisted breeding (Verma et al., 2022; Surya Krishna et al., 2023a).

Despite the crop’s large genome size and high ploidy level, transgenics have successfully improved biotic and abiotic stress tolerance (Nayyar et al., 2017; Ramasamy et al., 2021; Appunu et al., 2024; Chinnaswamy et al., 2024; Sharma et al., 2024). Particle bombardment and *Agrobacterium*-mediated transformation have been widely used for sugarcane genetic transformation (Surya Krishna et al., 2023a). Transformation efficiency of sugarcane is lower than most other crops and is affected by various factors such as explants, genotypes, transformation methods, and others (Verma et al., 2022). Red rot and viral diseases pose significant threats to sugarcane production and affect juice quality (Surya Krishna et al., 2023a; 2023b). Transgenic events overexpressing the *β-1,3-glucanase* gene and *endochitinase* gene from *Trichoderma* spp. have shown resistance against the red rot pathogen infection (Nayyar et al., 2017; Sharma et al., 2024). Virus resistant events have been created through the coat protein genes of the sugarcane yellow leaf virus and sugarcane mosaic virus (Budeguer et al., 2021; Surya Krishna et al., 2023b). Additionally, *Bt* technology has been successfully employed in sugarcane to develop transgenic events resistant to sugarcane borers (Gao et al., 2016; Wang W. Z. et al., 2017; Cristofoletti et al., 2018; Dessoky et al., 2021). Many abiotic stress tolerance events have been developed, and the overexpression of transcription factors such as *TERF1* and *EaNF-YB2* showed improved drought stress tolerance (Rahman et al., 2021; Chinnaswamy et al., 2024). The improved proline and antioxidant activity achieved the improved stress tolerance in these transgenic lines and effective management of hydrogen peroxide and MDA under water deficit conditions (Rahman et al., 2021; Chinnaswamy et al., 2024). Next to drought, salinity stress is a significant abiotic stressor that affects sugarcane production. Overexpression of genes such as *EaGly III* (Mohanan et al., 2021) and *EaALDH7* (Appunu et al., 2024) have been proven to enhance sugarcane resilience to salinity stress. Furthermore, transgenic events overexpressing the sugarcane *G-protein-coupled receptor (ShGPCR1)* exhibited tolerance to multiple abiotic stresses, such as drought, salinity, and cold (Ramasamy et al., 2021). GPCRs play a crucial role in plant stress tolerance by acting as molecular switches that transmit signals upon exposure to drought, salinity, and temperature extremes, to the cellular machinery (Ramasamy et al., 2021; Majumdar et al., 2023). Upon activation, G-proteins interact with various effectors and secondary messengers, regulating the expression of stress-responsive genes, including those coding for antioxidative enzymes and osmoprotectants, thus leading to acquired stress tolerance (Ramasamy et al., 2021; Majumdar et al., 2023).

Despite the complex genetic nature of sugarcane, genome editing has been successful in inducing targeted mutations in multiple alleles of marker genes such as *magnesium chelatase subunit I (MgCh)* (Eid et al., 2021) and *acetolactate synthase (ALS)* genes (Oz et al., 2021). Laksana et al. (2024) successfully produced genome-edited lines with low lignin content, making them suitable for second-generation bioethanol production by editing the *SoLIM* transcription factor. CRISPR/Cas9 genome editing of multiple copies of the *LIGULELESS1* (*LG1*) gene resulted in tunable leaf angle phenotypes, and the mutants showed a high biomass yield and tillers under field conditions (Brant et al., 2024). Despite the complexity of sugarcane’s genome, genetic engineering and gene editing have tremendous potential to accelerate the breeding process, as recent reports demonstrate its success in improving various traits (Surya Krishna et al., 2023a; Kumar et al., 2024; Brant et al., 2025). The application of transgenics and genome editing techniques for sugarcane crop improvement is given in Table 4.

**TABLE 4:** Transgenic and genome editing approaches for sugarcane crop improvement.

S. No	Genetic engineering technique	Gene of interest	Improved trait	References
1	Transgenics	*Cp* gene	Resistance against sugarcane yellow leaf virus	Zhu et al. (2011)
2	Transgenics	*AVP1*	Tolerance to salinity	Kumar et al. (2014)
3	Transgenics	*SMV- CP* genes	Sorghum mosaic virus	Guo et al. (2015)
4	Transgenics	*EPSPS*	Tolerance to Glyphosate	Noguera et al. (2015), Wang W. Z. et al. (2017)
5	Transgenics	*EaHSP70*	Enhanced Drought and salinity tolerance	Augustine et al. (2015b)
6	Transgenics	*EaDREB2*	Improved Drought and salinity tolerance	Augustine et al. (2015a)
7	Transgenics	*SbALS*	Tolerance to chlorsulfuron	Dermawan et al. (2016)
8	Transgenics	*β-1,3-glucanase*	Resistance to red rot disease	Nayyar et al. (2017)
9	Transgenics	*CaneCPI-1*	Reduced sugarcane weevil infestation	Schneider et al. (2017)
10	Transgenics	*siRNAs2* and *siRNA4*	Resistance against sugarcane mosaic virus	Aslam et al. (2018)
11	Transgenics	*cry1Ac*	Enhanced Resistance against Sugarcane Borer	Gao et al. (2016), Zhou et al. (2018), Dessoky et al. (2021)
12	Transgenics	*Bacillus thuringiensis* derived *Vip3A*	Enhanced Resistance against Sugarcane stem Borer	Riaz et al. (2020)
13	Transgenics	SCMV-CP genes	Enhanced resistance to SCMV infection	Yao et al. (2017), Apriasti et al. (2018), Widyaningrum et al. (2021)
14	Transgenics	*V-ATPase E*	Reduced sugarcane weevil infestation	Mohan et al. (2021)
15	Transgenics	*EaEXPA1*	Improved drought tolerance	Narayan et al. (2021)
16	Transgenics	*SoACLA-1*	Enhanced drought tolerance	Zhu et al. (2021)
17	Transgenics	*EaGly III*	Salinity Tolerance	Mohanan et al. (2021)
18	Transgenics	*ShGPCR1*	Tolerance to drought, salinity, and cold stresses	Ramasamy et al. (2021)
19	Transgenics	*SoTUA*	Tolerance to cold	Chen et al. (2021)
20	Transgenics	*Pac1*	Tolerance to sugarcane streak mosaic virus	Wang et al. (2022)
21	Transgenics	*Pinellia pedatisecta Agglutinin PPA*	Resistance to Sugarcane Woolly Aphid	Zhao et al. (2022)
22	Transgenics	*OsBSR1*	Resistance to sugarcane smut	Maeda et al. (2023)
23	Transgenics	*Small Ubiquitin-Like Modifier protease OTS1*	Tolerance to drought	Masoabi et al. (2023)
24	Transgenics	*Endochitinase*	Resistance to red rot disease	Sharma et al. (2024)
25	Transgenics	*Cry1Ac*	Resistance against giant Borer	Sakuno et al. (2024)
26	Transgenics	*EaALDH7*	Improved salinity tolerance	Appunu et al. (2024)
27	CRISPR/Cas9	*SoLIM*	Reduced lignin content	Laksana et al. (2024)
28	CRISPR/Cas9	*LIGULELESS1 (LG1)*	Tuneable leaf angle phenotype and improved biomass yield	Brant et al. (2024)

## 6 Current challenges and future directions

Crop improvement through inter and intraspecific crosses and genome-assisted breeding has been promising in crop plants (Ahmar et al., 2020; Gaikwad et al., 2020). Despite their huge success in diploids and seed-propagated crops, it has been highly challenging to obtain the same success in polyploids due to the complex genetic architecture. Polyploids have multiple homologous or homeologous copies of each chromosome, resulting in multiple alleles at each genetic locus, making it difficult to track the introgressed genes or to precisely recover the genetic background of the recipient parent during backcrossing (Jiang et al., 2000; Heslop-Harrison et al., 2023). The segregation of traits in polyploids does not follow simple mendelian ratios due to polysomic inheritance (in autopolyploids) or disomic inheritance with sub-genome interactions (in allopolyploids) (Feldman and Levy, 2009; Parisod et al., 2010; Leal-Bertioli et al., 2018). This makes it notably more challenging to recover the target trait or maintain heterozygosity for the polygenic traits. During introgression, repeated backcrossing may lead to loss of desirable heterozygosity, negatively affecting agronomic performance and adaptability. On the other hand, cross-compatibility barriers, such as low fertility in hybrids, hybrid sterility, incomplete pairing of homologous chromosomes, or failure of chromosome doubling, genomic instability, and epigenetic modifications, make the situation even more complicated (Birchler et al., 2005; Comai, 2005; Osabe et al., 2012; Te Beest et al., 2012; Soares et al., 2021; Healey et al., 2024). Considering the complexities associated with utilizing traditional breeding methods for polyploid crop improvement, genetic engineering serves as a viable alternative for the targeted improvement of vegetatively propagated polyploids for various agronomic traits, including yield, quality, and stress resistance (Kamthan et al., 2016; Gao, 2021; Marone et al., 2023). The vegetative propagation allows for clonal replication of desirable GE mutants and transgenic events, preserving traits without the segregation and variation associated with sexual reproduction (Schaart et al., 2021). This unique advantage makes genetic engineering techniques more promising for polyploid breeding. However, it also has some drawbacks, such as the need for field gene banks for germplasm maintenance, pathogen accumulation in plant material, and the increased susceptibility of monocultures to pest or disease epidemics (Chahal and Gosal, 2002; Acquaah, 2015).

Although the clonal propagation allows the fixation of desirable alleles in a single generation, when it comes to removing the Cas9 and other transgenes from the desirable GE mutants leaving the mutation unaffected is an arduous task. Transformation techniques leading to genetically modified plants reduce public acceptance of targeted mutagenized (edited) plants (Nguyen et al., 2023). However, the issue of stable transgene integration can be addressed by transient transformations with ribonucleoprotein complexes (RNPs) that degrade rapidly once they enter the cell and possess less off-target effects (Gu et al., 2021; He et al., 2022; Surya Krishna et al., 2023a). Scoring GE mutants is challenging in polyploid genomes as multiple mutation types can occur within a single gene, as each gene copy may harbor different mutations in the targeted region. In addition, the presence of somaclonal variation in the GE mutants could make the evaluation of genome-editing lines challenging (Fossi et al., 2019; Li et al., 2019; Graham et al., 2020). The somaclonal variation is more prevalent in the protoplast-generated plants (Fossi et al., 2019; Li et al., 2019). The other significant challenges are lower transformation efficiency, genotype dependency, lower editing efficiency, low *in vitro* regeneration ability, and more off-target effects, underscoring the need for genotype-independent, multiplex, and DNA-free delivery systems (May et al., 2023). Some of the important factors that significantly impact GE efficiency are guide RNA selection, promoters for guide RNA and Cas expression and crop specific codon optimization of the Cas gene. The most commonly used promoter to drive Cas9 expression in potato genome editing is the CaMV35S (Kusano et al., 2018; Nakayasu et al., 2018; Enciso-Rodriguez et al., 2019; Kieu et al., 2021; Bi et al., 2023; Karlsson et al., 2024; Norouzi et al., 2024). Similarly, CaMV35 is also used in strawberry (Gao et al., 2020; Feng et al., 2021), banana (Kaur et al., 2020; Tripathi et al., 2021; Ntui et al., 2024) and sugarcane (Brant et al., 2024). However, other promoters such as pUbi (Shao et al., 2019; Xing et al., 2020; Chen et al., 2023), PcUbi (Tripathi J. N. et al., 2019; López-Casado et al., 2023), AtUBQ (Zhou et al., 2021), and PPDK (Johansen et al., 2019; Lucioli et al., 2022) have also been used for Cas9 expression. For guide RNA expression in these crops, endogenous U6 promoters or U6 promoters from rice and *Arabidopsis* are commonly used.

Several techniques based on PCR and sequencing are available to detect mutations in the GE lines. PCR based approaches such as PCR-RFLP, T7E1, heteroduplex mobility assays (HMA), Competition-based PCR, high-resolution melting analysis, and digital droplet PCR enable the rapid preliminary screening of GE mutants in an inexpensive and rapid way (Jung and Altpeter, 2016; Lomov et al., 2019; Camerlengo et al., 2020). However, these techniques only provide information about the presence or absence of a mutation but fail to indicate the type of mutation. The sequencing of the targeted loci in GE mutants provides detailed information about the types of mutations, namely, base substitutions or Insertions and Deletions (INDELs) and the allelic nature of the genes targeted. Generally, the sanger sequencing of amplicons of the targeted regions has been carried out to identify the nature and frequency of the induced mutation (Grohmann et al., 2019; Yun et al., 2022). Despite the advantages of sanger sequencing, multiple alleles of the polyploids complicate the interpretation of sequencing results, as it leads to overlapping peaks in the electropherogram, making it difficult to read the sequence accurately (Zischewski et al., 2017; Cardi et al., 2023). Polyploids often have heterozygous mutations spread across different allelic copies, which makes it difficult to resolve heterozygous loci using sanger sequencing (Griffin et al., 2011). In polyploids, one or two mutant alleles may be overshadowed by multiple copies of the wild-type alleles. This lowers the mutant allele frequency in the sequencing signal, making it hard to detect point mutations. However, next-generation sequencing technologies address the limitations associated with sanger sequencing by offering better resolution and coverage, enabling them to detect mutations in multiple alleles as well as off-target mutations (Griffin et al., 2011; Zischewski et al., 2017; Motazedi et al., 2018; Li et al., 2019; Cardi et al., 2023). For instance, Brant et al. (2024) used CRISPR Amplicon Next-Generation Sequencing to identify the mutation in the multiple alleles of *LIGULELESS1* gene is sugarcane. Similarly, Martín-Pizarro et al. (2019) sequenced the GE mutants of strawberry for *TM6* MADS-box gene using high-throughput paired-end amplicon sequencing. In the case of transgenics, transgene integration is random in the genome. PCR-based screening followed by southern blot hybridization enables the identification of transgenic events with stable transgene integration (Liu Y. et al., 2017; Widyaningrum et al., 2021; Vennapusa et al., 2022; Jiang et al., 2023; Umesha et al., 2025). However, the next-generation sequencing of transgenic events allows us to find out the exact location of the transgene integration as well as the gene copy numbers (Park et al., 2017; Xu et al., 2024). It also enables us to study the potential off-target effects based on the site of the transgene integration, whether in the gene-rich or non-genic regions of the genome. Despite the progress made in the crops discussed in this review, genome editing remains under-deployed in other vegetatively propagated polyploids such as sweet potato, yam, and taro, which are important food crops in some tropical countries (Divya et al., 2024; Tripathi et al., 2024a). Transgenics have been successful in improving potato, banana, strawberry, and sugarcane for biotic and abiotic stress tolerance, nutritional content, and quality. In contrast, genome editing has shown notable progress in improving these crops for disease resistance. However, genome editing has shown few improvements in insect resistance, abiotic stress tolerance, and post-harvest applications. The complex regulatory networks involved in stress response and the highly complex nature of these traits hinder the improvement in abiotic stress tolerance ability by targeted editing of one or a few genes in these crops (Zhang et al., 2022; Kumar et al., 2023).

## 7 Genetically engineered crops and regulations

Transgenic events and site-directed nuclease (SDN) events are the two outcomes of genetic engineering techniques that enable the targeted crop improvement for a particular trait. Transgenic events involve the integration of foreign DNA into a plant genome to express novel traits that are not present in the gene pool of a crop species. Classic examples of commercialized transgenic crops are potatoes, sugarcane, cotton, corn, canola and soybean, with the first two (potato and sugarcane) being vegetatively propagated polyploids (Castagnola and Jurat-Fuentes, 2012; Koul et al., 2024; ISAAA, 2025). While transgenic technologies have successfully introduced significant improvements in crop traits, the foreign DNA from different organisms has led to public skepticism and stringent regulatory oversight, particularly regarding environmental and food safety concerns in many parts of the world (Lucht, 2015; Smyth, 2017; Koul et al., 2024).

**FIGURE 2:**
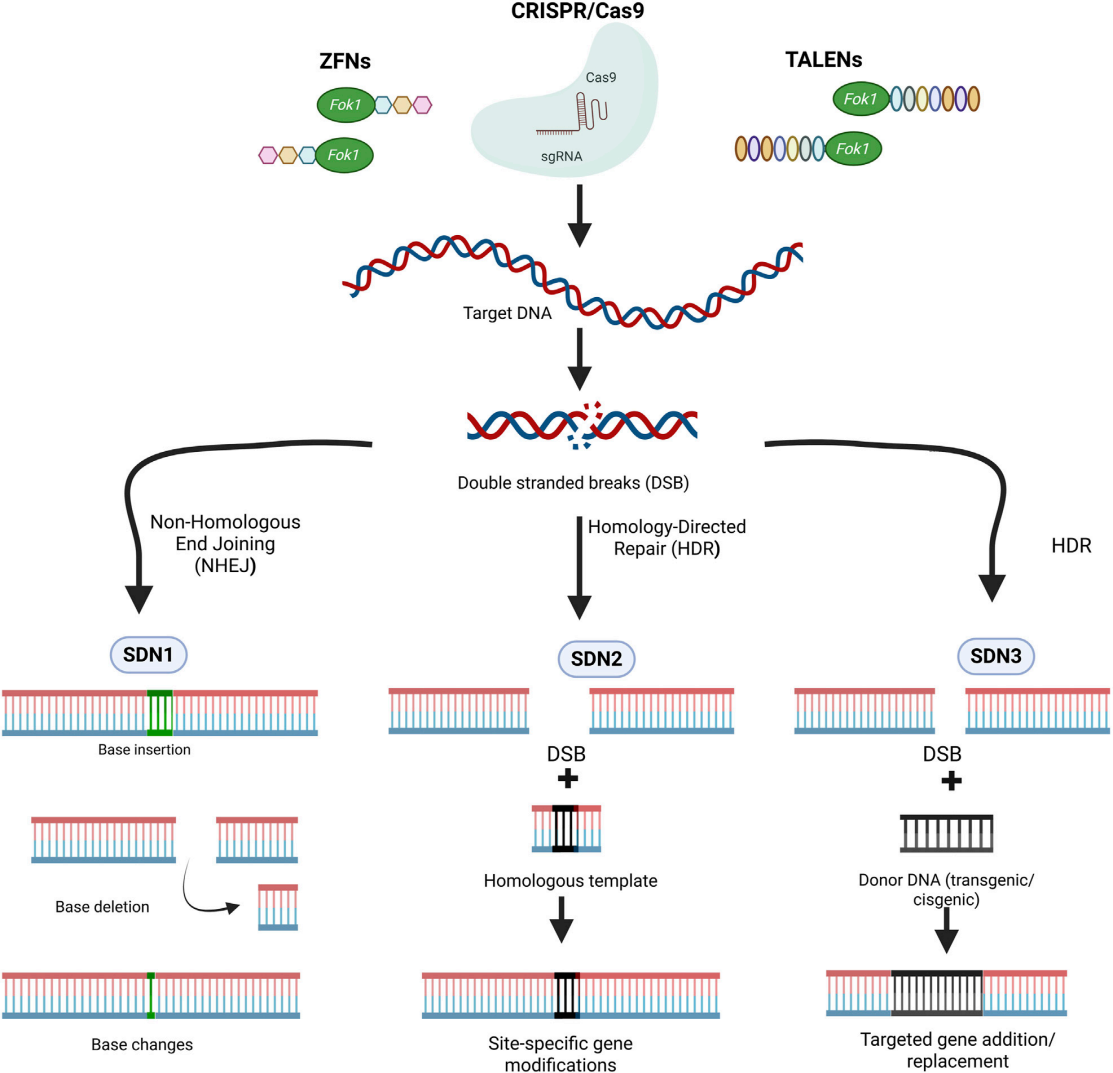
Classification of SDN (Site-Directed Nucleases) products for genome editing based on the nature of genomic modifications. (ZFN, Zinc Finger Nuclease; TALENs, Transcription Activator-Like Effector Nucleases; CRISPR/Cas9, Clustered Regularly Interspaced Short Palindromic Repeats/CRISPR-associated protein 9). Image created using Biorender (https://BioRender.com).

On the other hand, genome-editing tools such as CRISPR/Cas9, TALENs (Transcription Activator-Like Effector Nucleases), and ZFNs (Zinc Finger Nucleases) make targeted modifications at specific genomic loci, offering higher accuracy and reduced off-target effects. SDN products are classified into three main categories SDN 1, SDN 2, and SDN 3 based on the nature of the genomic modifications (Figure 2) (Sprink et al., 2016; Zannoni, 2019). SDN-1 involves small base deletions insertions or substitutions at the target site by harnessing the power of non-homologous end joining (NHEJ) without the use of foreign DNA template (Sprink et al., 2016; Zannoni, 2019). SDN-2 introduces precise nucleotide substitutions or modifications using short donor DNA templates (Sprink et al., 2016; Zannoni, 2019). The products of SDN-1 and SDN-2 are transgene-free and indistinguishable from natural mutations. SDN-3 enables the site-specific integration of a long stretch of DNA sequences, producing plants similar to transgenics or cisgenics (Sprink et al., 2016; Zannoni, 2019). Genome-edited crops such as high oleic acid soybean, less bitter mustard greens, and high GABA tomato are on the market, offering consumers products with enhanced qualities achieved through precise genetic modifications (Koul et al., 2024; Genetic Literacy Project, 2025). As a result, SDN1-based crops are subject to less stringent regulations in many jurisdictions, leading to wider acceptance among the public and policymakers.

Regulations for new breeding techniques (NBTs) are rapidly evolving worldwide. GMO’s are primarily regulated in two ways as process-based regulation and product-based regulation (Sprink et al., 2016). Product-based regulations are followed by countries such as the United States, Canada, Japan, and a few others, where the guidelines and rules focus on the safety, efficacy, and impact of the final GMO products rather than the process used to create them (Sprink and Wilhelm, 2024; Genetic Literacy Project, 2025). Process-based regulation is followed by countries such as India, Australia, Brazil, and most others where the guidelines and rules focus on the techniques and methods used to create the GMOs rather than solely on the characteristics of the final product (Sprink and Wilhelm, 2024; Genetic Literacy Project, 2025). The United States, Canada, China, Japan, Brazil, and the Philippines have approved the SDN-1 GE events for commercial cultivation, and some other countries are under field trials (Koul et al., 2024; Sprink and Wilhelm, 2024; Genetic Literacy Project, 2025). Despite the commercial success of genome-edited seed crops like tomato, soybean, and mustard, the market success of vegetatively propagated GE mutants like potatoes, sugarcane, and bananas is yet to be seen (Koul et al., 2024; Genetic Literacy Project, 2025). The legal environment surrounding genome-edited plants presents a multifaceted challenge, with diverse national approaches impacting the pace and direction of research and innovation (Metje-Sprink et al., 2020; Koul et al., 2024; Sprink and Wilhelm, 2024).

## 8 Public perception of genetically engineered crops

Consumer attitudes towards these technologies exhibit significant variability due to limited public understanding, a lack of trust in information sources, and ethical and societal concerns (Wunderlich and Gatto, 2015; Cui and Shoemaker, 2018; Woźniak-Gientka et al., 2022; Medani et al., 2024). A primary concern is the potential for unintended health consequences, such as allergies, toxicity, or long-term health effects (Cui and Shoemaker, 2018; Woźniak-Gientka et al., 2022; Catherine et al., 2024). While extensive research has generally shown genetically modified (GM) foods to be safe, these concerns persist (Atherton, 2002; Hallman et al., 2013). Concerns include the potential for horizontal gene flow to wild plants, the impact on biodiversity, and the development of herbicide-resistant superweeds and pesticide residues in the plant products (Bawa and Anilakumar, 2013; Vennapusa et al., 2018; Gbashi et al., 2021; Medani et al., 2024). Some individuals have ethical concerns about interfering god’s creation by manipulating the genetic makeup of organisms and dependence on industries for seeds (Hossain and Onyango, 2004; Frewer et al., 2013; Scott et al., 2018; Medani et al., 2024). Many consumers lack trust in regulatory agencies, the food industry, and even scientists regarding GMOs (Ghasemi et al., 2013; Turker et al., 2013; Akbari et al., 2019). This lack of trust stems from past controversies, conflicting information, and perceived industry bias (Dean and Shepherd, 2007; Sorgo et al., 2012; Scott et al., 2016; Cui and Shoemaker, 2018; Robayo-Avendaño et al., 2018).

Public adoption of GM crops discussed in this review varied notably based on the socio-economic status, health benefits, environmental attributes, and awareness of GM technology (Kikulwe et al., 2011; Muringai et al., 2020; Vermeulen et al., 2020). A survey among Canadian consumers regarding the acceptance of gene-edited versus genetically modified potatoes showed that consumers preferred gene-edited products over GM products (Muringai et al., 2020). Some consumers are even interested in paying more for health benefits such as low acrylamide content when fried and for positive environmental attributes (Muringai et al., 2020). A study on South African consumers’ perception and acceptance of sugar made from genetically modified sugarcane showed that the acceptance reduced with socio-economic status and age (Vermeulen et al., 2020). Those who support GM sugar were willing to pay a higher premium amount, especially when environmental benefits were emphasized (Vermeulen et al., 2020). A consumer survey on the attitudes and perceptions of GM bananas in Uganda showed that over 90% of respondents agreed they would purchase GM bananas if they offered better taste or nutrition and were priced the same as conventional bananas (Kikulwe et al., 2011). However, acceptance dropped to 39% if GM bananas were priced higher than non-GMOs (Kikulwe et al., 2011). A study on consumer preference towards specialty labelled products among Utah consumers revealed that non-GMO labeling of strawberry provides tangible market advantages through consumer willingness to pay premiums, even if modest (Curtis et al., 2024). The consumer preference pattern suggests that non-GMO strawberries will likely maintain competitive advantages over GM alternatives, particularly when combined with other valued attributes like organic certification or local production (Curtis et al., 2024). The willingness to pay premiums for non-GMO labeling suggests underlying consumer concerns about genetic modification.

Bridging the gap between scientific consensus and public perception of GM technologies requires clear communication, transparency, and inclusive engagement (Gbashi et al., 2021; Woźniak-Gientka et al., 2022; Catherine et al., 2024). Simplifying concepts through multimedia tools and integrating biotechnology into education can foster understanding and trust (Gbashi et al., 2021; Catherine et al., 2024; Medani et al., 2024). Highlighting GM success stories, such as improved yield and nutritional qualities and reduced pesticide use, provides concrete proof of their benefits. Community outreach, open dialogues on ethics and safety, and leveraging trusted voices like scientists and farmers can address misconceptions and build confidence (Gbashi et al., 2021; Woźniak-Gientka et al., 2022; Catherine et al., 2024; Medani et al., 2024). These strategies can collectively enhance public trust and facilitate the responsible adoption of GM technologies. Furthermore, the global regulatory policy environment for GE crops (non-transgenics) is still emerging. This evolving landscape can shape innovation in crop improvement and influence its overall socioeconomic benefits to society (Schaart et al., 2021; Kalaitzandonakes et al., 2023; Lakhani et al., 2023).

## 9 Conclusion

While transgenics remain valuable for introducing novel genes and complex traits, genome editing has emerged as an efficient and precise platform for genome modifications, often with fewer regulatory hurdles. Both technologies enable targeted modifications in complex genomes, effectively overcoming traditional breeding challenges associated with ploidy levels and asexual reproduction in vegetatively propagated crops. By focusing on key traits such as biotic and abiotic stress resistance, yield improvement, and quality enhancement, genetic engineering and genome editing can significantly accelerate the development of superior cultivars in vegetatively propagated polyploid crops. As evidenced by past achievements, these approaches can be used to breed crops with desirable traits in minimal time compared to conventional breeding methods. Overall, transgenic and genome editing technologies play complementary roles in breeding clonally propagated polyploids, offering promising solutions to meet future food security challenges under changing climate scenarios.
